# The glycolipid flocculosin-A from the fungus *Anthracocystis flocculosa*, or how to deal with cotton-wool-like crystals

**DOI:** 10.1107/S2052520625008583

**Published:** 2025-11-07

**Authors:** Thierry Prangé, Giang Nam Phan, William Shepard, Michel Ponchet, Laurent Lapeyre, Mohamed Mehiri

**Affiliations:** a CITCoM, CNRS UMR 8038, Faculté de Pharmacie, Université Paris Cité, 75006Paris, France; b CNRS UMR 7272Université Nice Côte d’Azur, 06108, France; c Synchrotron SOLEIL 91190Saint Aubin France; d INRAE 1355, CNRS UMR 7254Sophia Agrobiotech, 06903Sophia Antipolis, France; e Group Lapeyre Consultant, 96, Chemin de la Cardelle, 06530Peymeinade, France; Siberian Branch of Russian Academy of Science, Russian Federation

**Keywords:** flocculosin-A, *Anthracocystis flocculosa*, fungus, crystal growth

## Abstract

Flocculosin A is a strong antifungal metabolite secreted by the Basidiomycota fungus *Anthracocystis flocculosa* (formerly *Pseudozyma flocculosa*). It crystallizes in the form of long, soft cotton-wool-like needles, usually observed together with the mycelium in liquid culture conditions. Many crystallization tests were carried out. Among them, only a very slow cooling technique was able to yield suitable crystals, which were needle-like with a maximum thickness of 5 µm and several millimetres long. These crystals permitted us to record single-crystal X-ray diffraction data and solve the crystal structure as well as to determine the absolute configurations of the three chiral centres present in the two lipid chains.

## Abstract 2

Crystals of flocculosin-A, the major antifungus compound from *Anthracocystis flocculosa*, are optimised using a cyclic temperature control.

## 1. Introduction

*Anthracocystis flocculosa* is a basidiomycetous fungal yeast with powerful antagonistic activity against powdery mildews (Avis & Bélanger, 2002 ▸). For many years, this inhibitory activity had been believed to be mainly due to the production of a single glycolipid, flocculosin, whose 3D structure has been the subject of numerous investigations (Cheng *et al.*, 2003 ▸; Mimee *et al.*, 2005 ▸). Although a recent study showed that flocculosin is not associated to a biocontrol activity (Santhanam *et al.*, 2021 ▸), this compound and analogs are still an attractive research subject due to their broad spectrum of antimicrobial properties (Mimee *et al.*, 2005 ▸; Mimee *et al.*, 2009 ▸). Considerable interest has developed in flocculosin derivatives, not only for their strong fungicidal activities, but also for evaluation as future economic sources of ω-hydroxy fatty acids (Becker *et al.*, 2020 ▸) and surfactants molecules (Paulino *et al.*, 2017 ▸). It is interesting to note that many other strains of basidiomycete yeast produce cellobiose lipids, including ustilagic acid analogues (Kulakovskaya *et al.*, 2005 ▸), which can also be exploited as a source of new active molecules.

In order to fully understand the structure–activity relationships of flocculosins, we performed a study of isolation and structure elucidation of the leading compound, flocculosin-A, whose structural scaffold consists of a cellobiose disaccharide with different degrees of acetyl­ation, linked to a hy­droxy-palmitoic acid and a hy­droxy-caproic acid via glycosidic bonds. However, the structure of flocculosin-A poses a serious challenge because the product always crystallizes as very long, curved, soft thin needles, less than 5 µm thick and of poor crystallinity.

## 2. Experimental

### 2.1. Materials

HPLC-grade solvents and reagents were purchased from Sigma-Aldrich (Quentin-Fallavier, France). Vacuum-liquid chromatography (VLC) was performed using C18 (50–70 mesh) resin obtained from Sigma–Aldrich. Column chromatography was conducted on Merck silica gel (70–230 mesh). Thin layer chromatography was performed on glass pre-coated silica gel 60 F254 plates (Merck, Darmstadt, Germany).

Product characterizations: NMR spectra were recorded on a Bruker AV spectrometer (400 MHz for ^1^H and 100 MHz for ^13^C). High-resolution mass spectra were obtained on a Thermo Instruments ESI-MS system connected to a Thermo Instruments HPLC system.

### 2.2. Isolation and purification of flocculosin-A

The fungus *Anthracocystis flocculosa* was isolated on carnation leaves grown in INRAE greenhouses (Ponchet, 1988 ▸). It was cultured on glucose asparagine medium (Picard *et al.*, 2000 ▸). Ten (1 L) Erlenmeyer flasks containing 200 ml of medium were inoculated with 500 µL of a suspension of yeast-like bodies (10^6^ propagules per ml). The cultures were maintained under constant agitation (80 rpm) in the dark for four days.

The fully fermented cultures were filtered to separate the culture filtrate from the mycelium.

Two litres of the culture medium were applied to C18 VLC (10 × 10 cm) and eluted with successive CH_3_OH/H_2_O mixtures (25:75, 50:50, 80:20, 100:0, each ∼0.8 L) followed by 0.8 L of a CH_2_Cl_2_/CH_3_OH mixture (50:50) to yield five fractions (M1–M5). Fraction M3 (4.5 g) was pure flocculosin-A (compound 1). The other fractions contain minor compounds or isomers. For example, fraction M2 (223 mg), purified by silica gel column chromatography (1.5 × 60 cm), and eluted with three ternary mixtures CHCl_3_/EtOH/H_2_O (7:1:0.1, 6:1:0.1, 5:1:0.1) yielded besides additional flocculosin-A (71 mg), two other derivatives named flocculosin-B (25 mg) and flocculosin-C (18 mg). All in all, seven parent derivatives bearing the same cellobiose moiety, either intermediates or precursors with various substitutions were isolated in addition to the major compound flocculosin-A (named flocculosin-B to -H). They will be described elsewhere.

Flocculosin-A was obtained as an amorphous powder. Molecular formula: C_40_H_70_O_19_. High-resolution electron spray ionization mass spectrometry = 853.4474 [M−H]^−^ (calcd for C_40_H_69_O_19_^−^ 853.4439) = 855.4577 [M+H]^+^ (calcd for C_40_H_71_O_19_^+^ 855.4584).

### 2.3. Crystallization of flocculosin-A

Solvents were first distilled, filtered through fritted glass No. 5, and stored in a cold place (4°C).

Flocculosin-A is sparingly soluble in water, moderately in hy­droxy­lated solvents, and very soluble at high temperatures in lipophilic solvents such as chloro­form, acetone, *etc*. All attempts to get crystals by temperature variations in all of these solvents, and mixtures thereof, led invariably to cotton-wool-like fibres, unsuitable for diffraction studies.

Obtaining fibres is usually the result of a large difference in crystal growth along three perpendicular directions. Flocculosin-A is in this case with a lateral/longitudinal growth ratio of 1:1000. The result is fibres several millimetres long and only a micron in diameter. To solve this problem and improve this ratio, we adopted a method of cyclic temperature variation between partial crystal dissolution (50°C) and complete crystallization (−10°C) over a slow time variation in a suitable solvent, compound TFT (tri­fluoro-toluene, CF_3_-C_6_H_5_) is considered to be a substitute for chloro­form or di­chloro­methane. Due to the thermal stability of flocculosin-A, the procedure that led to exploitable crystals was as follows.

A solution of flocculosin-A (10 mg in 1 ml of TFT) in a small glass vials (2 mL) was heated to 100°C until complete dissolution occurred. The vials were isolated from the exterior with silicon stoppers and placed in a dewar filled with 200 ml of hot (100°C) NaCl brine, acting as a temperature buffer. The dewar was then enclosed in a large polystyrene box also containing a large volume of hot brine. The closed box was allowed to cool down at room temperature (the internal temperature was followed by an external thermocouple reader fixed to the box). The temperature equilibrium was achieved within a full day. The box was then placed in a freezer and cooled down to −10°C for half a day. The crystallization of flocculosin-A was checked at this stage under a microscope equipped with a polarizer. This completed the first temperature cycle.

Afterwards, two more temperature cycles were made, *i.e.* for each, heating the box up to 50°C to partially dissolve the needles, and cooling it again at room temperature, then down to −10°C. It was expected that the dissolution/recrystallization of the needles will be mostly along the fastest growing axis than along the slowest, and that crystal growth will favour increasing the thickness of the needles after recrystallization, because of the physical limitation of the needle’s elongation in the small cylinder of crystallization. Each cycle was completed over one or two days.

**Figure 1:**
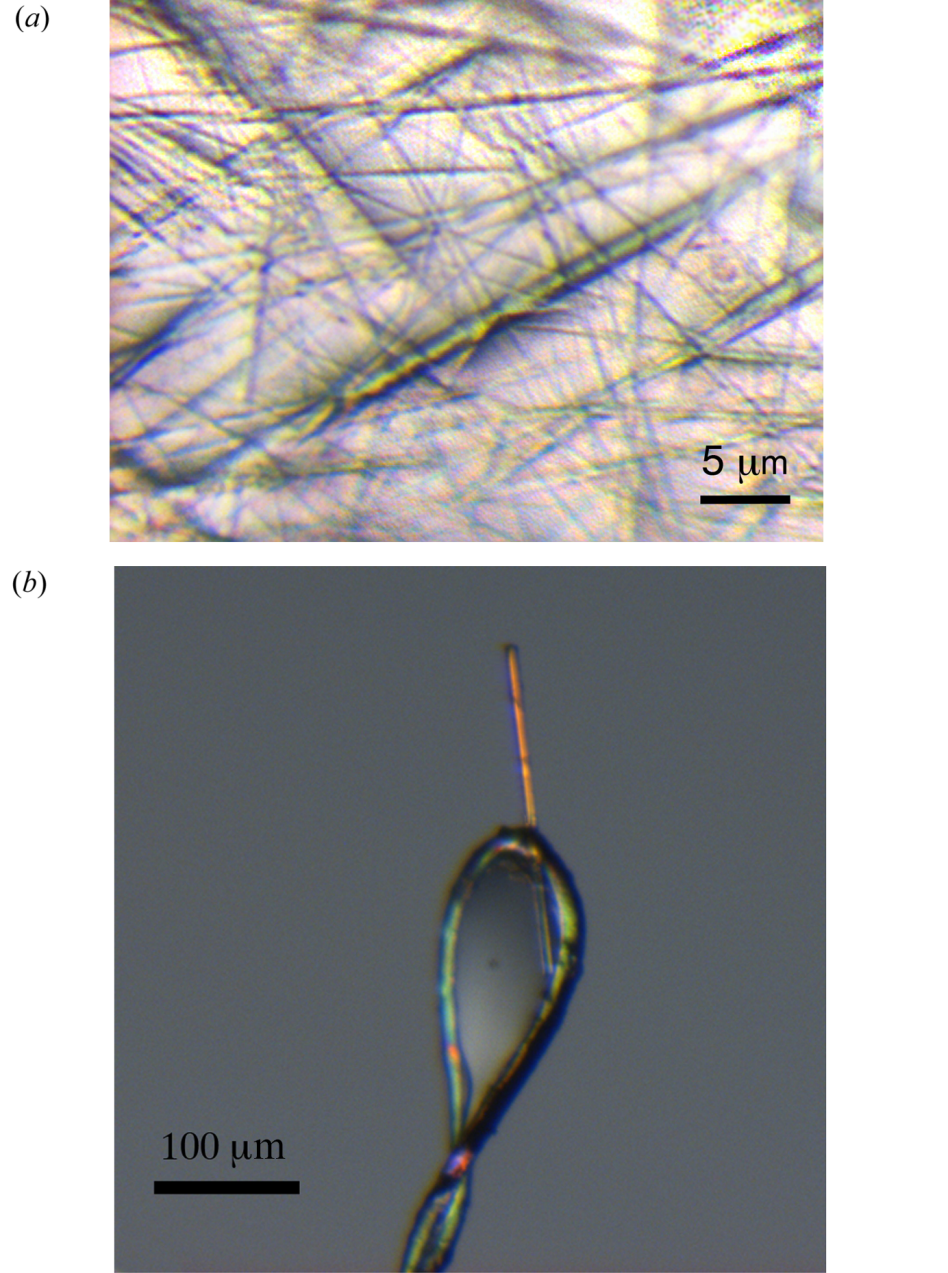
(*a*) Crystals of flocculosin-A. (*b*) A 3 × 3 × 200 µm crystal mounted on a cryo-loop.

At the end of the third cycle, needles of flocculosin-A were harvested, dispersed on a glass plate under a microscope, with a few drops of perfluoro-polyether Mw4500 (Alfa-Aesar, Karlsruhe, Germany) over them [Fig. 1 ▸(*a*)]. Several selected needles, 3 to 5 µm thick, the maximum we observed, were selected, cut at about 200 µm in length and fished out with a Hampton cryo-loop, being careful to avoid any twisting or bending of the crystal due to the surface tension of the drop [Fig. 1 ▸(*b*)]. They were immediately frozen in liquid nitro­gen.

### 2.4. X-ray data collection

X-ray diffraction data were recorded at synchrotron SOLEIL (St Aubin, France) on the micro-focus beamline PROXIMA 2A (Duran *et al.*, 2013 ▸). The X-ray wavelength was set at λ = 0.872 Å. The quality of diffraction differs greatly along the needle axis. A good position was selected from a small number of X-ray diffraction frames, showing an acceptable diffraction quality that can be characterized by a good mosaicity and no elongation of Bragg spots. The X-ray diffraction data were recorded in the standard rotation method using a DECTRIS EIGER X 9M pixel detector. The data were processed with the *XDS* software (Kabsch, 2010 ▸), and formatted for the *SHELX* suite of programs (Sheldrick, 2008 ▸).

## 3. Results and discussion

**Figure 2:**
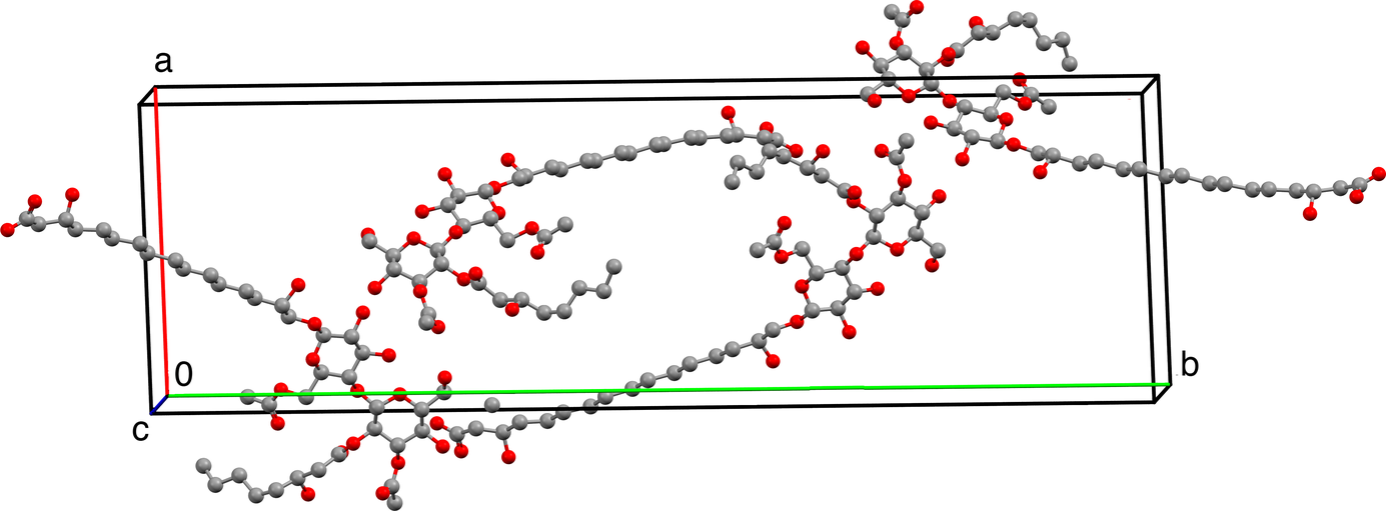
*Mercury* (Macrae *et al.*, 2006 ▸; 2020 ▸) view of the flocculosin-A packing along the shortest axis. The two palmitoic and caproic chains are along the *c* axis, leading to a strongly dissymmetric cell. Water and ethanol solvate molecules have been removed for clarity.

The crystal structure of flocculosin-A was solved by direct methods (*SHELXS*) and refined (*SHELXL*) with individual isotropic, then anisotropic thermal factors for the O atoms. H atoms were introduced at theoretical positions but were not refined. Crystal data and refinement statistics are reported in Table 1 ▸. In the final refined structure, the carboxyl­ate group of the palmitoic chain was found disordered and refined into two positions, roughly perpendicular to each other (Fig. 2 ▸). As flocculosin-A was initially isolated from an ethano­lic extract, the crystal structure interestingly retains one water (ordered) and one (disordered and partially occupied) molecule of ethanol in its crystal packing.

**Figure 3:**
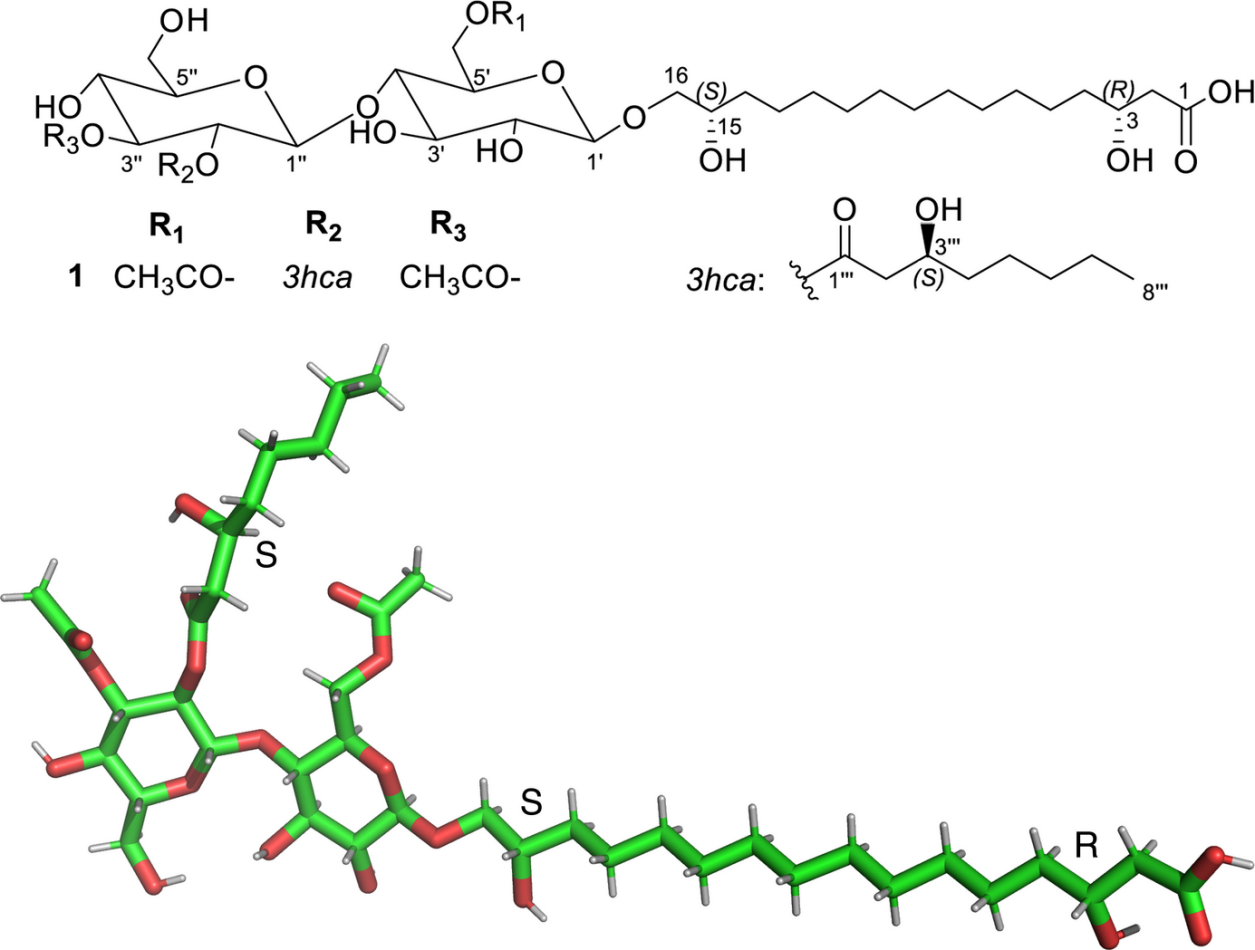
The chemical formula of flocculosin-A (top) and a *pyMOL* (Schrödinger & DeLano, 2020 ▸) view (bottom) of the molecule with H atoms in theoretical positions (not refined).

The complete structure of flocculosin-A is built around an acetyl­ated cellobiose moiety containing two lipid chains (Fig. 3 ▸). The characteristic of these two chains is a hy­droxy­lation whose absolute configuration was unknown. Due to the known absolute configuration of the cellobiose moiety [formerly β-d-glucopyran­osyl(1→4) d-glucopyran­ose], the absolute configuration of the three chiral centres of the lipidic chains–two in the palmitoic chain and one in the caproic chain–we can now confidently extrapolate from that of the glucose moieties, as shown in Fig. 3 ▸.

## 4. Conclusion

By adopting a special protocol of crystallization employing slow cyclic temperature variations, we were able to improve the quality and size of the crystalline fibres of flocculosin-A. Its structure was solved by X-ray diffraction, which makes it possible to determine by comparison the absolute configuration of the asymmetric C atoms of the lipid chains attached to the cellobiose moiety of the molecule. The evaluation of the anti-fungal properties of flocculosin-A and its analogs are underway.

## Supplementary Material

Crystal structure: contains datablock(s) floc. DOI: 10.1107/S2052520625008583/tq5025sup1.cif

Structure factors: contains datablock(s) I. DOI: 10.1107/S2052520625008583/tq5025Isup2.hkl

CCDC reference: 2295682

## Figures and Tables

**Table 1:** Experimental details

Crystal data	
Chemical formula	C_40_H_70_O_19_·C_2.64_H_5.96_O_1.66_
*M*_r_	894.97
Crystal system, space group	Orthorhombic, *P*2_1_2_1_2
Temperature (K)	103
*a*, *b*, *c* (Å)	16.72 (1), 54.08 (1), 5.49 (1)
*V* (Å^3^)	4964 (10)
*Z*	4
Synchrotron, λ (Å)	0.872
μ (mm^−1^)	0.09
Crystal size (µm)	3 × 3 × 200

Data collection	
No. of measured, independent and observed [*I* > 2σ(*I*)] reflections	3646, 3646, 2986
θ_max_ (°)	29.9
(sin θ/λ)_max_ (Å^−1^)	0.571

Refinement	
*R*[*F*^2^ > 2σ(*F*^2^)], *wR*(*F*^2^), *S*	0.083, 0.204, 1.54
No. of reflections	3646
No. of parameters	668
No. of restraints	13
H-atom treatment	H atoms treated by a mixture of independent and constrained refinement
(Δ/σ)_max_	0.845
Δρ_max_, Δρ_min_ (e Å^−3^)	0.36, −0.32

Computer program: *SHELXL2018/3* (Sheldrick, 2018 ▸).
